# A Novel Micro Cold Atmospheric Plasma Device for Glioblastoma Both In Vitro and In Vivo

**DOI:** 10.3390/cancers9060061

**Published:** 2017-05-30

**Authors:** Zhitong Chen, Hayk Simonyan, Xiaoqian Cheng, Eda Gjika, Li Lin, Jerome Canady, Jonathan H. Sherman, Colin Young, Michael Keidar

**Affiliations:** 1 Department of Mechanical and Aerospace Engineering, The George Washington University, Washington, DC 20052, USA; zhitongchen@gwu.edu (Z.C.); xiaoqian@gwmail.gwu.edu (X.C.); egjika@gwu.edu (E.G.); lilin@email.gwu.edu (L.L.); 2 Department of Pharmacology and Physiology, The George Washington University, Washington, DC 20052, USA; hayksimonyan@email.gwu.edu; 3 Jerome Canady Research Institute for Advanced Biological and Technological Sciences, US Medical innovation LLC, Takoma Park, MD 20912, USA; drjcanady@canadysurgicalgroup.com; 4 Department of Neurosurgery, The George Washington University, Washington, DC 20052, USA; jsherman@mfa.gwu.edu

**Keywords:** micro-sized plasma device, glioblastoma, reactive oxygen species, reactive nitrogen species, cancer therapy

## Abstract

Cold atmospheric plasma (CAP) treatment is a rapidly expanding and emerging technology for cancer treatment. Direct CAP jet irradiation is limited to the skin and it can also be invoked as a supplement therapy during surgery as it only causes cell death in the upper three to five cell layers. However, the current cannulas from which the plasma emanates are too large for intracranial applications. To enhance efficiency and expand the applicability of the CAP method for brain tumors and reduce the gas flow rate and size of the plasma jet, a novel micro-sized CAP device (µCAP) was developed and employed to target glioblastoma tumors in the murine brain. Various plasma diagnostic techniques were applied to evaluate the physics of helium µCAP such as electron density, discharge voltage, and optical emission spectroscopy (OES). The direct and indirect effects of µCAP on glioblastoma (U87MG-RedFluc) cancer cells were investigated in vitro. The results indicate that µCAP generates short- and long-lived species and radicals (i.e., hydroxyl radical (•OH), hydrogen peroxide (H_2_O_2_), and nitrite (NO_2_^−^), etc.) with increasing tumor cell death in a dose-dependent manner. Translation of these findings to an in vivo setting demonstrates that intracranial µCAP is effective at preventing glioblastoma tumor growth in the mouse brain. The µCAP device can be safely used in mice, resulting in suppression of tumor growth. These initial observations establish the µCAP device as a potentially useful ablative therapy tool in the treatment of glioblastoma.

## 1. Introduction

Plasma is an ionized gas consisting of positive/negative charges, radicals, neural atoms and ultraviolet (UV) photons, that is, a gaseous matter with quasi-neutral charges [1,2,3]. Atmospheric plasma at or near ambient temperature has led to a new field of plasma medicine [4,5], and cold atmospheric plasma (CAP) has attracted a lot of attentions due to its remarkable potential to affect biological processes [2,6]. In this context, the potential of CAP in diverse bio-medical applications has been explored, including disinfection, wound treatments, control of inflammation, blood coagulation, cancer therapy, and regenerative medicine [7,8,9,10,11]. The efficacy of CAP in the proposed applications relies on the synergistic action of the reactive oxygen species (ROS), reactive nitrogen species (RNS), free radicals, UV photons, charged particles, and electric fields [12,13,14,15]. A low dose of ROS and RNS was reported to induce cell proliferation as well as cell death, while a high dose of ROS/RNS can damage proteins, lipids, DNA, and induce apoptosis [16,17,18,19]. Some results also indicated that exposing cancer cells to CAP resulted in the production of free radicals that could cause apoptotic cell death [20]. Many studies of CAP for cancer therapy have indicated that CAP does not harm normal tissue when applied at the appropriate dosages [21,22,23,24]. Taken together, CAP therapy has been introduced as a cost effective, rapid and selective treatment modality for killing cancer cells.

CAP may be generated by a range of different plasma devices such as the plasma jet, dielectric barrier discharge, corona discharge, and gliding arc [25,26,27,28]. CAP-generated plasma can be directly applied to skin cancers. However, most cancers occur inside the body, therefore plasma irradiation is not practical due to the high voltage, the formation of discharge in the organ, gas delivery and plasma probe volume [29]. The biological effects of micro-sized plasma were studied in vitro and in vivo earlier [29,30,31]. However, these studies were not completely successful in carrying out CAP treatment at a specific position inside the body of animals. In this regard, glioblastoma is a highly malignant aggressive neoplasm of the primary central nervous system characterized by rapid growth, extensive angiogenesis, and resistance to all current therapies [32,33]. The major limitations of glioblastoma tumor treatment and eventual tumor recurrence are: (1) that the brain is susceptible to damage with conventional therapies; (2) tumor cells are very resistant to conventional therapies; (3) many drugs cannot cross the blood–brain barrier to act on brain tumors; and (4) the brain has a very limited capacity to repair itself [32,34]. Thus, it is necessary to develop a plasma device with a reduced gas flow rate and size, which can potentially be used in vivo by circumventing the problems listed above. Here, a new micro-sized CAP (µCAP) device employing helium gas was developed, and the effect of µCAP on glioblastoma both in vitro and in vivo was evaluated.

## 2. Results

### 2.1. µCAP and Optical Spectrum

**Figure 1:**
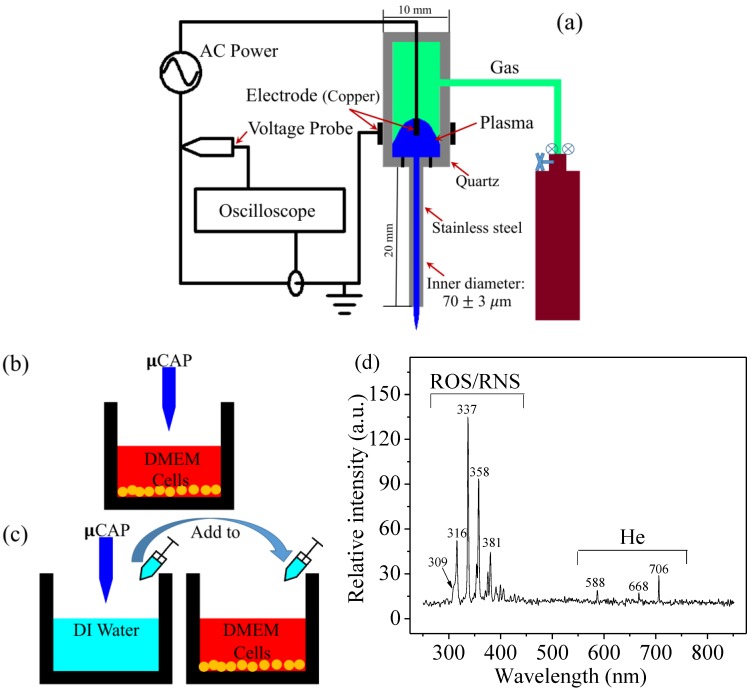
(**a**) Schematic representation of the micro-sized cold atmospheric plasma setup; (**b**) µCAP direct treatment: µCAP was directly applied to cells in DMEM (100 µL); (**c**) µCAP indirect treatment: DI water was treated with µCAP and then applied to cells in DMEM (70 µL DMEM + 30 µL treated DI water); (**d**) Optical emission spectrum detected from the He µCAP using UV-visible-NIR, in the 250–850 nm wavelength range. ROS: reactive oxygen species; RNS: reactive nitrogen species; CAP: cold atmospheric plasma; µCAP: micro-sized CAP; DMEM: Dulbecco’s Modified Eagle Medium; DI: Deionized; µCAP: Micro-sized cold atmospheric plasma; AC: Alternating current.

Figure 1a illustrates the µCAP setup used in this work. Figure 1b illustrates µCAP direct treatment in which the µCAP was used to treat culture medium containing glioblastoma cancer cells. Figure 1c shows µCAP indirect treatment in which the µCAP was used to first treat deionized (DI) water. After treatment, the DI water was transferred to culture medium containing cells (70 µL Dulbecco’s Modified Eagle Medium (containing cells) + 30 µL treated DI water, total 100 µL). The times for both direct and indirect treatment were 5, 10, 30, 60, and 120 s. Figure 1d shows the spectrum of the He µCAP with a flow rate of 0.1 L/min. The identification of the emission line and bands was performed mainly according to [35]. The peaks of 315 nm, 337 nm, 357 nm, and 380 nm represent the photon emission intensity as the results of excited N_2_ drops from the state C^3^Π_u_ to B^3^Π_g_ with different upper and lower vibration quantum numbers. The 281-nm peak represents the photon emitted by excited NO drops from A^2^Σ^+^ to X^2^Π [35]. The helium bands were assigned between 500 and 750 nm as shown in Figure 1d. Species at a wavelength of 309 nm could be defined as OH. Both He bands between 250 nm and 425 nm could be defined as ROS/RNS. The nitrogen and OH emission bands likely arise from the helium supply and ambient air.

### 2.2. Electron Density of He µCAP Jet

**Figure 2:**
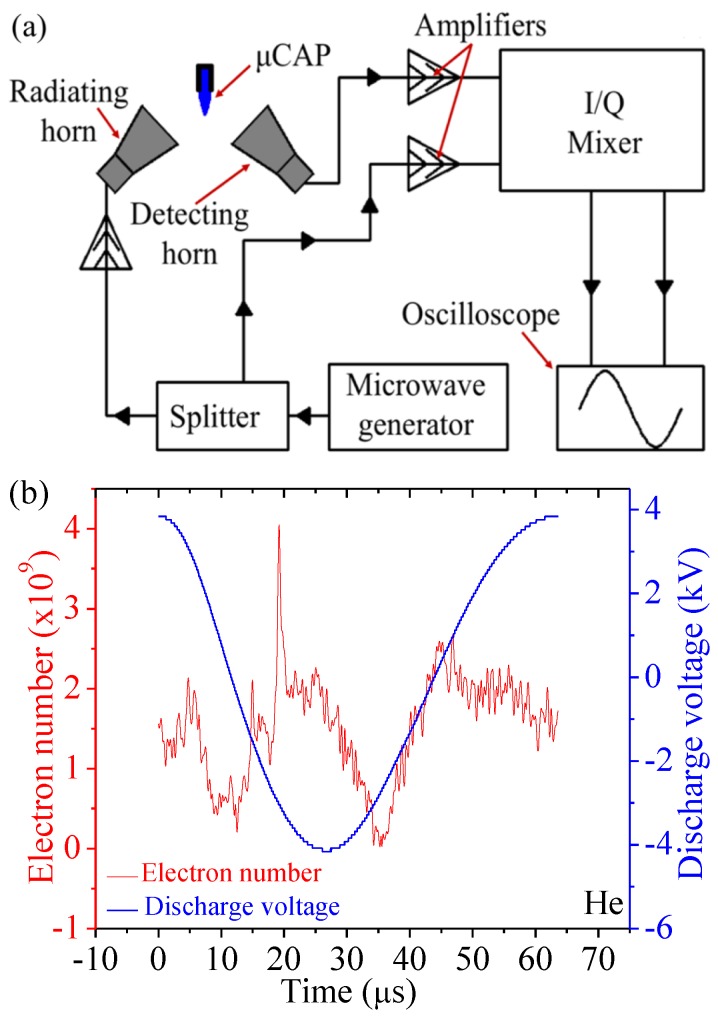
Schematics of the Rayleigh microwave scattering (RMS) experimental setup (**a**), as well as the electron number and discharge voltage of He (**b**). He: Helium.

The experimental Rayleigh microwave scattering (RMS) system is shown in Figure 2a. The dependence of the output RMS signal on parameters of the scattering channel can be expressed as U=AσV, where A is the proportionality coefficient ($A=263.8\;V\Omega /cm^{2}$) and V is the plasma volume. The volume of the plasma column was determined from intensified charged-coupled device (ICCD) images. σ is plasma conductivity using the following expression: $\sigma =2.82\times 10^{-4}n_{e}v_{m}/\left(\omega^{2}+v_{m}^{2}\right)$, $\Omega^{-1}cm^{-1}$, where $v_{m}$ is the frequency of the electron-neutral collisions, $n_{e}$ is the plasma density, and ω is the angular frequency [36,37]. Combining these equations yields $n_{e}=\left(\left(\omega^{2}+v_{m}^{2}\right)U\right)/\left(\left(2.82\times 10^{-4}Av_{m}\right)V\right)$. Therefore, we can calculate the total electron number in the plasma as $N_{e}=n_{e}V=U\left(\omega^{2}+v_{m}^{2}\right)/\left(2.82\times 10^{-4}Av_{m}\right)$. The electron number of He µCAP is presented in Figure 2b, and the total electron number for one discharge period is $2.4\times 10^{9}$.

### 2.3. Detection of RNS Generated by µCAP

**Figure 3:**
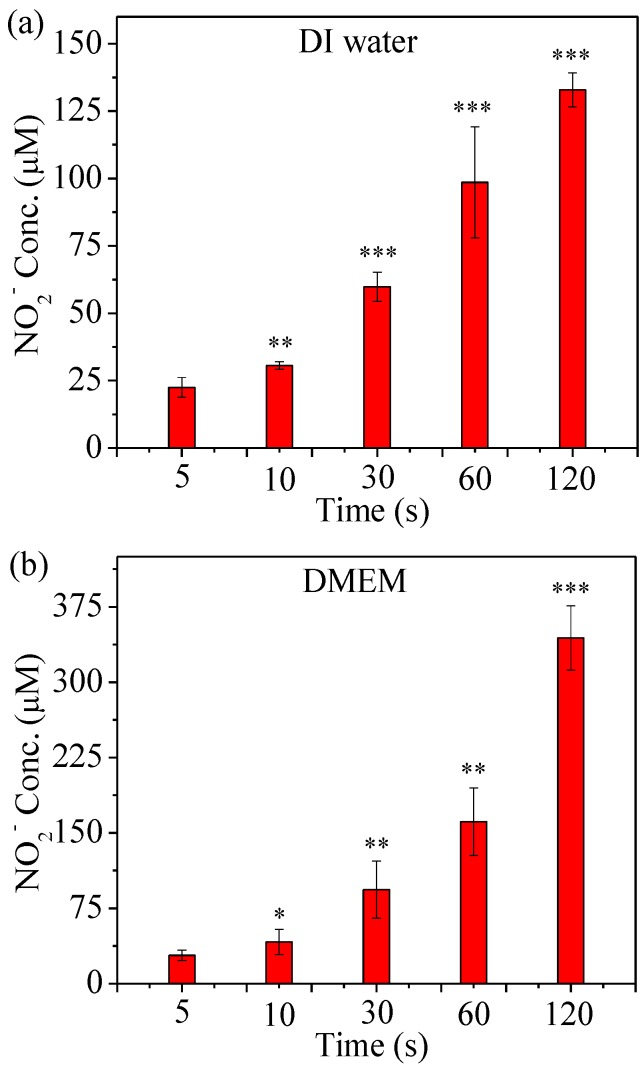
RNS concentration in He µCAP-treated DI water (**a**) and DMEM (**b**). Student t-test was performed, and the statistical significance compared to µCAP 5 s treatment is indicated as * *p* < 0.05, ** *p* < 0.01, *** *p* < 0.001. (*n* = 3).

µCAP treatment of DI water and Dulbecco’s Modified Eagle Medium (DMEM) were performed to induce changes in the concentration of ROS and RNS as a function of the treatment time. Indeed, as shown in Figure 3, the NO_2_^−^ concentration in He µCAP-treated DI water and DMEM increases with treatment duration. The NO_2_^−^ mainly originates as NO (N_2_ + e → 2N + e, N + O_2_ → NO + O, 4NO + O_2_ + 2H_2_O → 4NO_2_^−^ + 4H^+^) [38,39,40], while most of NO is formed in the gas phase during the afterglow a few milliseconds after the discharge pulse. The NO_2_^−^ concentration in DMEM treated with He µCAP is higher than that in DI water (*p* < 0.05 for all treatment times). On the other hand, DMEM comprises over 30 components such inorganic salts, amino acids and vitamins, while the effect of plasma irradiation on these compounds is still unknown. Plasma might react with amino acids to form NO_2_^−^, which provides a possible explanation for higher NO_2_^−^ concentration in DMEM than in DI water. Plasma-activated media produces a lot of species, including NO_3_^−^. In our previous study, the Ph range of plasma solutions was around 5.0 to 6.0 [16]. On the other hand, different concentrations of H_2_O_2_ show a Ph range from 4.5 to 6.2. Therefore, Ph of Μcap should be between 4.5 and 6.0.

### 2.4. Assess Relative Concentration of Hydroxyl Radical

**Figure 4:**
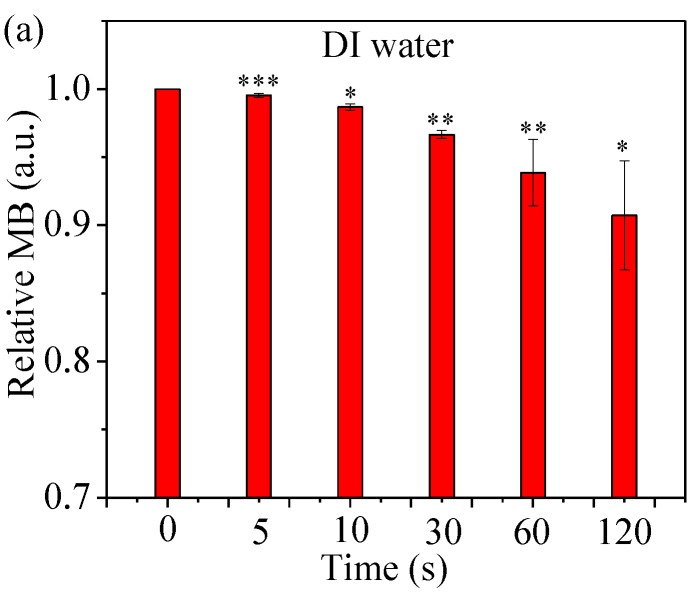

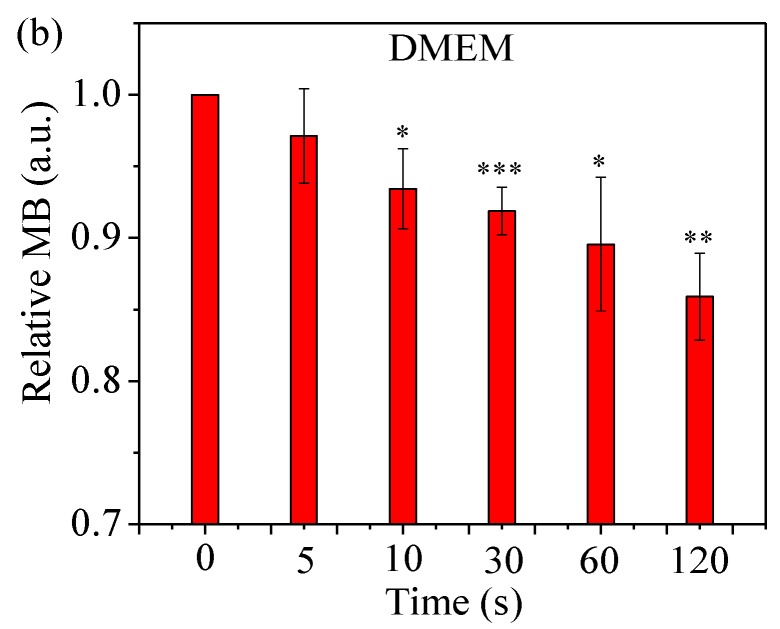
Relative methylene blue (MB) concentration for assessing the concentration of hydroxyl free radicals in He µCAP-treated DI water (**a**) and DMEM (**b**). Student *t*-test was performed, and the statistical significance compared to MB without µCAP treatment (0 s) is indicated as * *p* < 0.05, ** *p* < 0.01, *** *p* < 0.001. (*n* = 3).

Methylene blue (MB) was used to assess the relative concentration of hydroxyl radicals (•OH). It is known that MB reacts with •OH aqueous solutions, leading to a visible color change [41]. As shown in Figure 4, the relative MB concentration decreases with treatment time of He µCAP. The relative MB concentration decreases by 9.3% after He µCAP-treated DI water for 120 s. While this change is significant, He µCAP-treated DMEM showed a stronger degradation action (14.7%), suggesting that more short-living reactive species are generated in DMEM. Plasma reacting with DMEM might form a new product that reacts with methylene blue, which may explain why He Μcap-treated DMEM showed a stronger degradation action than DI water. Overall, these findings demonstrate that there is an increase in the relative concentration of •OH as a function of µCAP treatment time.

### 2.5. Detection of ROS Generated by µCAP

**Figure 5:**
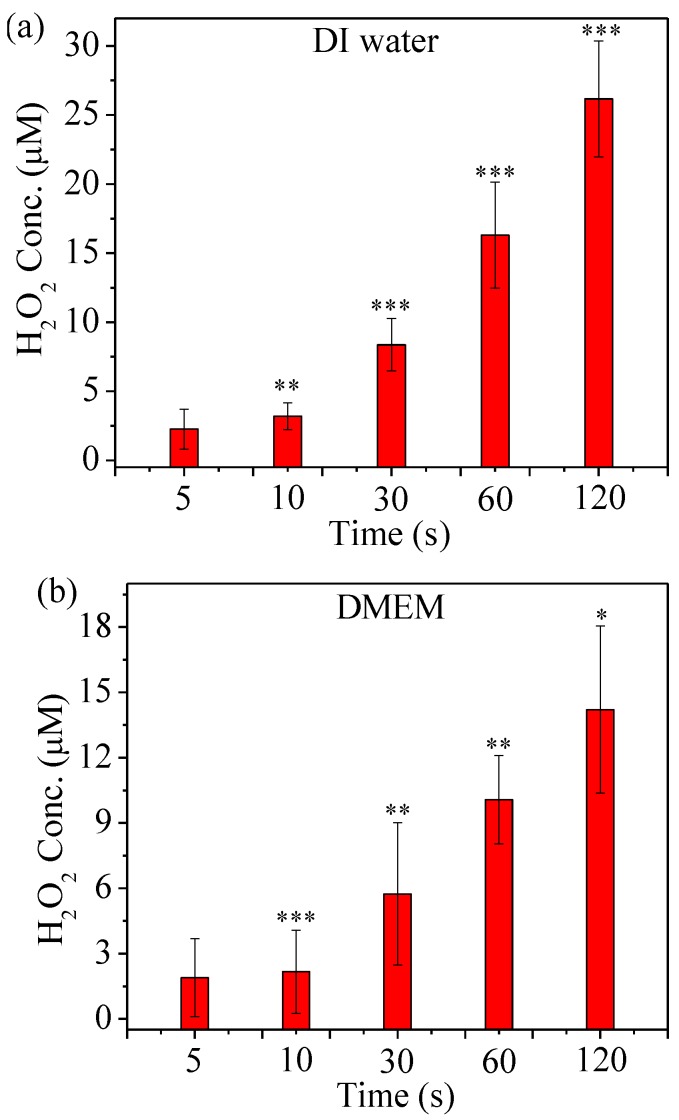
H_2_O_2_ concentration in µCAP-treated DI water (**a**) and DMEM (**b**). Student *t*-test was performed, and the statistical significance compared to µCAP 5 second treatment is indicated as * *p* < 0.05, ** *p* < 0.01, *** *p* < 0.001. (*n* = 3).

Figure 5 shows the ROS concentration dependence on treatment time in He µCAP-treated DI water and DMEM. H_2_O_2_ generation might be attributed to the high electron density and energy of the plasma (He → He^+^ + e, He^+^ + H_2_O → H_2_O^+^ + He, H_2_O^+^ + H_2_O → H_3_O^+^ + •OH; He + e → He * + e, He * + H_2_O → He + •OH + H∙; H_2_O + e → H_2_O * + e, H_2_O * → •OH + H∙; •OH + •OH → H_2_O_2_) [42,43]. The H_2_O_2_ concentration increases with treatment duration for both DI water and DMEM, although the concentration of H_2_O_2_ generated in DI water is higher than DMEM.

### 2.6. Cell Viability Follow In Vitro µCAP Treatment Duration

**Figure 6:**
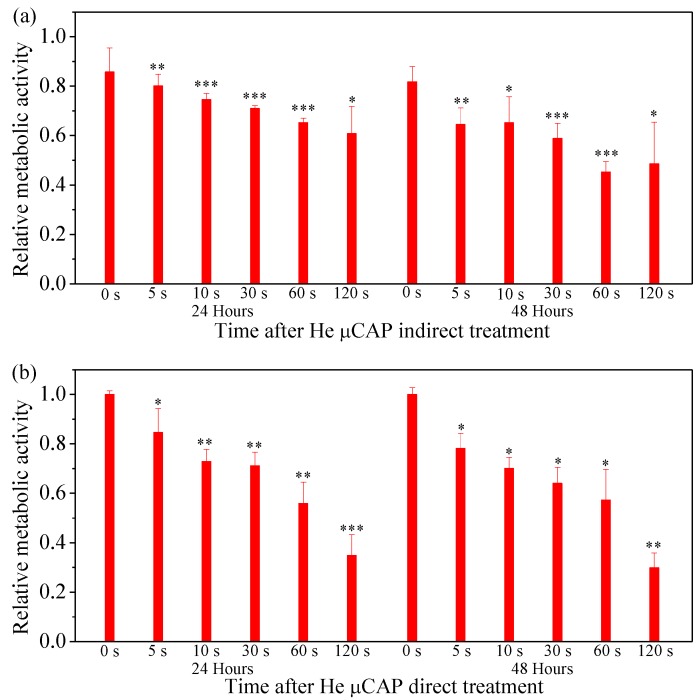
Cell viability of U87MG after 24 and 48 h of incubation with He µCAP indirect treatment (**a**) and direct treatment (**b**) for 0, 5, 10, 30, 60, and 120 s. The ratios of surviving cells for each cell line were calculated relative to controls (0 s) in DMEM. Student *t*-test was performed, and the statistical significance compared to cells present in DMEM (0 s) is indicated as * *p* < 0.05, ** *p* < 0.01, *** *p* < 0.001. (*n* = 3).

Figure 6a shows the cell viability of the glioblastoma cancer cells treated with He µCAP indirect treatment after 24 and 48 h. The cell viability after 24 h incubation (normalized by DMEM control) decreased by approximately 14.2%, 19.9%, 25.4%, 29.0%, 34.7%, and 39.2%, according to 0, 5, 10, 30, 60, and 120 s treatment durations, respectively. After 48 h of incubation, the cell viability decreased by approximately 18.3%, 35.6%, 34.8%, 41.1%, 54.7%, and 51.4%, for 0, 5, 10, 30, 60, and 120 s treatment durations, respectively. Figure 6b shows the cell viability of glioblastoma cancer cells after 24 and 48 h incubation with He µCAP direct treatment (treat 100 µL media containing cells) for 0, 5, 10, 30, 60, and 120 s duration. The cell viability of the glioblastoma cancer cells after 24-h incubation (normalized by control) decreased by approximately 15.4%, 27.1%, 28.9%, 44.1%, and 65.1%, according to 5, 10, 30, 60, and 120 s treatment durations, respectively. Similar dose-dependent (i.e., increase treatment time) decreases in cell viability were found with 48-h incubation.

### 2.7. In Vivo Targeting of Glioblastoma with µCAP

**Figure 7:**
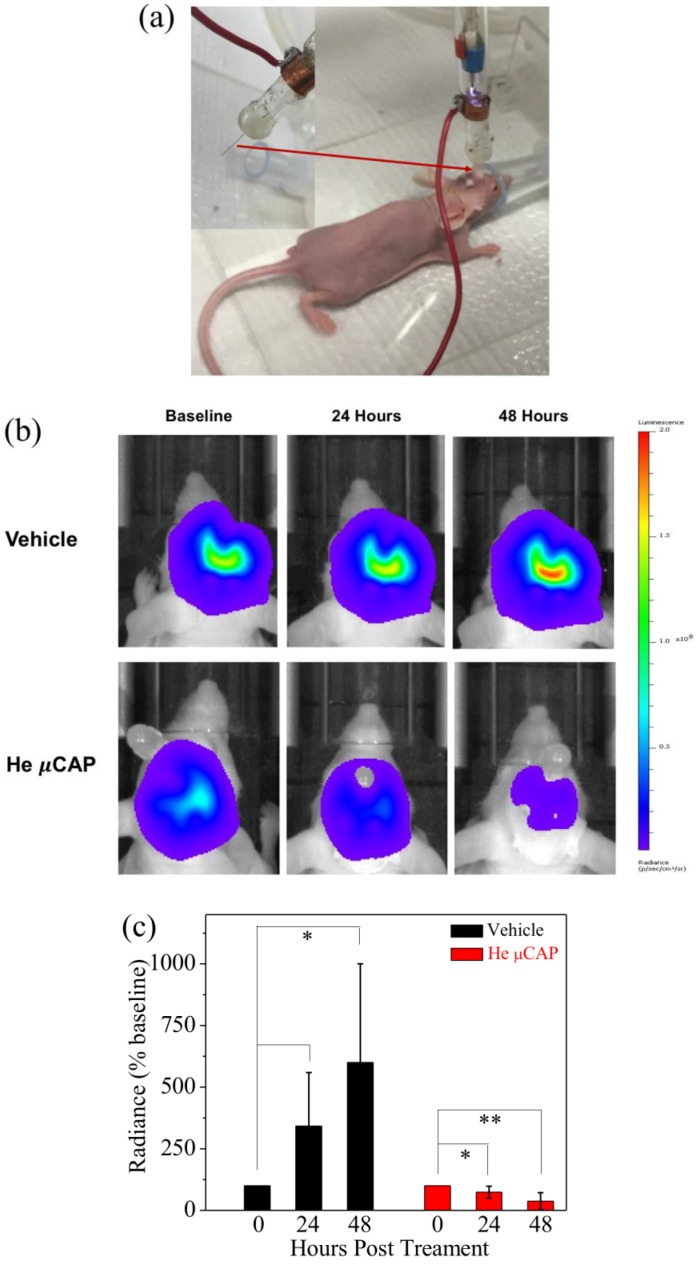
In vivo targeting of glioblastoma tumor with He µCAP. (**a**) Photograph of a new μCAP device for plasma delivery through an intracranial endoscopic tube to target glioblastoma tumors in the mouse brain; (**b**) Representative in vivo bioluminescence images illustrating glioblastoma tumor volume (i.e., light emission or radiance) at baseline and 2 days following He µCAP or vehicle (helium) treatment. Areas of high photon emission are shown in red and low are shown in blue; (**c**) Group summary data (*n* = 3/group) indicates that the tumor volume in control aggressively increased following treatment, whereas He µCAP delivery maintained tumor volume below basal levels.

In order to determine the effect of the novel plasma device effect on glioblastoma in vivo, we directly applied He µCAP for 15 s to glioblastoma tumors in the brain of living mice via an implanted endoscopic tube as shown in Figure 7a. Using in vivo bioluminescence imaging (Figure 7b), the tumor volume in a control animal (helium only) increased nearly 600% over the course of two days, whereas He µCAP-treated tumor volume decreased approximately 50% compared with baseline levels (Figure 7c). In the control animals, there is a clear increase in tumor volume over the 2-day period. In contrast, He µCAP-treated animals fell below baseline values. These striking findings demonstrate the potential of µCAP to inhibit glioblastoma tumor growth in vivo.

## 3. Discussion

CAP has received considerable attention for its potential biomedical applications. Emerging fields of application of CAP include wound healing, sterilization of infected tissue, inactivation of microorganisms, tooth bleaching, blood coagulation, skin regeneration, and cancer therapy. This has been collectively termed ‘plasma medicine’. Both direct and indirect applications of CAP have been shown to be effective for treating various cancer cells in vitro and in vivo [11]. However, as discussed above, the treatment of tumors deep within the body has been hampered by the limitations of CAP delivery tools. Thus, we aimed to develop and study how application of a novel He µCAP device could induce high cell death both in vitro and in vivo.

Treating culture media containing cells (direct treatment) was compared to treating DI water and removing it to culture media containing cells (indirect treatment). Our findings suggest that both He µCAP direct/indirect treatment could induce high cell death in glioblastoma cancer cells (Figure 6), although in general direct treatment was more effective than indirect treatment. Plasma contains the energy ions, free radicals, reactive species, UV radiation, and the transient electric fields inherent with plasma delivery, which interact with the cells and other living organisms. In many cases, it has been reported that plasma-induced apoptosis in cancer cells had no adverse effect on the normal cells when administered at the comparable dosage provided to cancer cells [11,44,45]. The absorption cross-section of ROS in the UV spectral range (10–400 nm) is relatively small [46], and the species lines between 300 and 350 are still not clearly determined. Radicals and electrons generated during plasma formation can be either short-lived or long-lived. These radicals or electrons reach a solution and form many complex reactions, which result in the formation of other short- and long-lived radicals or species. Short-lived radicals or species include superoxide (O_2_^−^), nitrite (NO), atomic oxygen (O), ozone (O_3_), hydroxyl radical (•OH), singlet delta oxygen (SOD, O_2_ (^1^∆g)), and peroxynitrite (ONOO^−^), etc. [47,48]. Long-lived species include hydrogen peroxide (H_2_O_2_) and nitrite (NO_2_^−^) [48].

In the case of indirect treatment µCAP (treatment of DI water and transfer to cells in a culture medium), one can argue that the primary effects are associated with long-lived species (H_2_O_2_ and NO_2_^−^). RNS are known to induce cell death via DNA damage, while ROS can induce cell death by apoptosis and necrosis [49,50]. When He is used as a carrier gas, the concentration of H_2_O_2_ and NO_2_^−^ in DI water rises with time (Figure 3 and Figure 5). Analyzing cell viability, one can see that He µCAP has a strong effect on glioblastoma (U87MG) cancer cells. The µCAP-generated H_2_O_2_ and NO_2_^−^ concentration increase with treatment time, in line with the linear increase in cancer cell killing efficiency. However, there might be additional antitumor pathways related to H_2_O_2_ and NO_2_^−^. For example, H_2_O_2_ and NO_2_^−^ are believed to generate peroxynitrite (H_2_O_2_ + 2NO_2_^−^ → 2HONOO^−^) which is known to be toxic to cells [51]. HONOO^−^ is stable at basic pH (DI water < 6) or otherwise it immediately decomposes into NO_3_^−^. However, HONOO^−^ may be difficult to generate in media because media is buffered and only slightly basic (pH > 7) [52]. On the other hand, aquaporins (AQPs) play a critical role in facilitating the passive diffusion of H_2_O_2_, especially AQP8 [45,53,54,55]. Not all AQPs can transport H_2_O_2_, and AQPs show various transportation degrees of H_2_O_2_ in different types of human tumors. AQP1, 4, 8, and 9 are highly expressed in glioblastoma cell lines [45,56]. Thus, the distinct expression pattern of AQP8 in glioblastoma might be responsible for cancer cells being sensitive to high H_2_O_2_ concentrations.

The effect of both short- and long-lived species or radicals is plausible when considering µCAP direct-treated glioblastoma cancer cells (in vitro, Figure 6) and tumors in the mouse brain (in vivo, Figure 7). Of note, DMEM comprises over 30 components such as inorganic salts, amino acids and vitamins and the effect of plasma irradiation on these compounds is still unknown. Similarly, the effect of plasma irradiation in murine brain is also unknown. Therefore, unknown reactive species or radicals could be generated during the irradiation of DMEM and during in vivo application. Short- and long-lived species or radicals in DMEM and mice brain containing •OH, NO, O_2_^−^, O, O_3_, O_2_ (^1^∆g), ONOO^−^, H_2_O_2_ and NO_2_^−^ are prominent components of antitumor plasma action [57]. The relative concentration of •OH in DMEM treated by He µCAP increases with treatment time (Figure 4). The color change of methylene blue shows the presence of OH radicals via immediate and distinct bleaching of methylene blue dye (qualitatively analysis) [41]. •OH-derived amino acid peroxides can contribute to cell injury because •OH itself and protein (amino acid) peroxides are able to react with DNA, thereby inducing various forms of damage [58,59]. When He µCAP activates DMEM, the concentration of both H_2_O_2_ and NO_2_^−^ rises with treatment time (Figure 3 and Figure 5), and synergism of H_2_O_2_ and NO_2_^−^ mentioned above might be an important factor. CAP also produces significant amount of ozone (O_3_), which is known to have strongly aggressive effect on cells [60]. Ozone has a role in the formation of biologically active ROS and RNS in aqueous media, which may be responsible for cell death [61,62]. Atomic oxygen (O) (including the ground state and all the excited states) is believed to have a significant effect on cells [63,64]. Superoxide (O_2_^−^) generated by plasma can activate mitochondrial-mediated apoptosis by changing mitochondrial membrane potential and simultaneously up-regulates pro-apoptotic genes and down-regulates anti-apoptotic genes for activation of caspases resulting in cell death [65]. Singlet delta oxygen O_2_ (^1^∆g) is another important ROS with the excitation energy of 0.98 eV. Highly reactive molecule O_2_ (^1^∆g) not only produces oxidative damage in many biological targets but is also a primary active species in the selective killing of tumor cells in the emerging cancer therapy [66,67]. Moreover, nitrite (NO_2_^−^) and superoxide (O_2_^−^) can easily form ONOO^−^ once they collide [68]. ONOO^−^ is a powerful oxidant and nitrating agent, which is known to be highly damaging to tumor cells [69]. Overall, the above results and discussion indicate that both direct and indirect routes of delivering CAP might be useful and should be considered in a clinical medical application. A further understanding of the precise underlying mechanisms will allow for determination of the “best” combination when used as a treatment strategy.

## 4. Materials and Methods

### 4.1. Experimental Device Configuration

In Figure 1a, the µCAP device consists of a two-electrode (copper) assembly with a central powered electrode and a grounded outer electrode wrapped around the outside of a quartz tube (10 mm). The electrodes were connected to the secondary output of a high voltage transformer. The peak-peak voltage was about 8 kV and the frequency of the discharge was around 15 kHz. The secondary output of the high voltage transformer was connected to the first input. The power of the first input is around 5 watts. At the end of a quartz tube, a 70 ± 3 µm inner diameter capillary tube (stainless steel) with a 20-mm length was attached and insulated by epoxy. The feeding gas for this study was industrial purity helium, which was injected into the quartz tube with a 0.1 L/min gas flow rate.

### 4.2. Optical Emission Spectroscopy (OES) Spectra Measurement

UV-visible-NIR, with a range of wavelength 200–850 nm, was investigated on plasma to detect various RNS and ROS (nitrogen [N_2_], nitric oxide [–NO], nitrogen cation [N^+2^], atomic oxygen [O], and hydroxyl radicals [–OH]). The spectrometer and detection probe were purchased from Stellar Net Inc. (Tampa, FL, USA). The optical probe was placed at a distance of 1.0 cm in front of the plasma jet nozzle. Data were collected with an integration time of 100 ms.

### 4.3. A Rayleigh Microwave Scattering System (RMS) for Electron Number Measurement

The experimental RMS system is schematically presented in Figure 2a. Two microwave horns were used for radiation and detection of microwave signal. Microwave radiation linearly polarized was scattered on the collinearly-oriented plasma channel and the scattered signal was then measured. The detection of the scattered signal was accomplished using a homodyne scheme by means of an *I/Q* mixer, providing in-phase (*I*) and quadrature (*Q*) outputs. For the entire range of scattered signals, the amplifiers and mixer were operated in linear mode. The total amplitude of the scattered microwave signal was determined by: $U=\sqrt{I^{2}+Q^{2}}$.

### 4.4. Cell Culture

Human glioblastoma cancer cells (U87MG, Perkin Elmer, Waltham, MA, USA) were cultured in Dulbecco’s Modified Eagle Medium (DMEM, Life Technologies, Washington, WA, USA) supplemented with 10% (v/v) fetal bovine serum (Atlantic Biologicals, Frederick, MD, USA) and 1% (v/v) penicillin and streptomycin (Life Technologies). Cultures were maintained at 37 °C in a humidified incubator containing 5% (v/v) CO_2_.

### 4.5. Determination of H_2_O_2_ Concentration

A fluorimetric hydrogen peroxide assay Kit (Sigma-Aldrich, St. Louis, MO, USA) was used for measuring the amount of H_2_O_2_, according to the manufacturer’s protocol. Briefly, 50 μL of standard curve, control, and experimental samples were added to 96-well flat-bottom black plates, and then 50 μL of Master Mix was added to each of well. The plates were incubated for 20 min at room temperature protected from light and fluorescence was measured by a Synergy H1 Hybrid Multi-Mode Microplate Reader at Ex/Em: 540/590 nm.

### 4.6. Determination of NO_2_^−^ Concentration

RNS levels were determined by using a Griess Reagent System (Promega Corporation, Madison, WI, USA) according to the instructions provided by the manufacturer. Briefly, 50 μL of samples and 50 μL of the provided sulfanilamide solution were added to 96-well flat-bottom plates and incubated for 5–10 min at room temperature. Subsequently, 50 μL of the NED solution was added to each well and incubated at room temperature for 5–10 min. The absorbance was measured at 540 nm by a Synergy H1 Hybrid Multi-Mode Microplate Reader.

### 4.7. •OH Accumulation in a Methylene Blue (MB) Solution

An MB solution was prepared by dissolving MB power in DI water and DMEM. MB solutions (100 μL per well, 0.01 g/L) in a 96-well flat-bottom black plate were treated by He µCAP for 5, 10, 30, 60, and 120 s. The gap between the outlet of µCAP and the surface of the samples was around 3 mm. As a control, two untreated MB solutions in triplicate were transferred to a 96-well flat-bottom black plate. The color change of methylene blue shows the presence of OH radicals via immediate and distinct bleaching of methylene blue dye (qualitatively analysis). Color change of MB solution was measured as the absorbance at 664 nm by a Synergy H1 Hybrid Multi-Mode Microplate Reader.

### 4.8. Cell Viability Following µCAP Indirect Treatment In Vitro

U87 cells were plated in 96-well flat-bottom microplates at a density of 3000 cells per well in 70 μL of complete culture medium. Cells were incubated for 24 h to ensure proper cell adherence and stability. On day 2, 30 μL of DI water was treated by He µCAP for 0, 5, 10, 30, 60, and 120 s, and was added to cells. Cells were further incubated at 37 °C for 24 and 48 h. The cell viability of the glioblastoma cancer cells was measured for each incubation time point with a 3-(4, 5-dimethylthiazol-2-yl)-2,5-diphenyltetrazolium bromide (MTT) assay. A volume of 100 μL MTT solution (Sigma-Aldrich) was added to each well followed by a 3-h incubation. The MTT solution was discarded and 100 μL per well of MTT solvent (0.4% (v/v) HCl in anhydrous isopropanol) was added to the wells. The absorbance of the purple solution was recorded at 570 nm with a Synergy H1 Hybrid Multi-Mode Microplate Reader.

### 4.9. Cell Viability Following µCAP Direct Treatment In Vitro

U87 cells were plated in 96-well flat-bottom microplates at a density of 3000 cells per well in 100 μL of complete culture medium. Cells were incubated for 24 h to ensure proper cell adherence and stability. On day 2, the cells were treated by He µCAP for 0, 5, 10, 30, 60, and 120 s. Cells were further incubated at 37 °C for 24 and 48 h. An MTT assay was used to assess cell viability as described above.

### 4.10. In Vivo Application of µCAP to Target Intracranial Glioblastoma

All animal protocols were approved by the George Washington University Institutional Animal Care and Use Committee. Eight-week-old female athymic nude mice (Charles River, NU(NCr)-Foxn1^nu^) were anesthetized intraperitoneally (i.p.) (Ketamine (100 mg/kg) mixed with Xylazine (10 mg/kg)) and placed in a stereotaxic frame. The surface of the skull was visualized with a dissecting microscope and horizontally leveled between bregma and lambda. A small hole was drilled at the desired location. U87MG-RedFluc cells (Perkin Elmer), containing a red-shifted firefly luciferase that allows for in vivo monitoring of tumor growth via light emission, were resuspended in DMEM at a concentration of 5 × 10^5^ cell/µL and injected into the frontal lobe at the following coordinates (relative to Bregma) using a Hamilton syringe: 2.2 mm ventral from the dorsal surface of the skull, 1.0 mm caudal, and 2.0 lateral. Then, 5 × 10^5^ cells were injected at a depth of 2.2 mm and the syringe was then retracted to 1.8 mm and an additional 5 × 10^5^ cells were then administered. To allow for delivery of µCAP, an endoscopic tube was then implanted at a depth of 2.2 mm and secured in place with dental cement. Mice were allowed to recover for 7 days and He µCAP or vehicle control (He alone) was then administered. In brief, mice were anesthetized with isofluorane and CAP was applied via the implanted endoscopic tube at 5-s intervals, followed by 15 s off, for a total CAP treatment time of 15 s. The jet was removed from the endoscopic tube to allow for venting of He. Tumor size was estimated using in vivo bioluminescent imaging before and for up to 48 h post CAP or vehicle treatment. For bioluminescent imaging, animals were anesthetized with isoflurane and the substrate luciferin was administered i.p., (150 mg/kg). The mice were then transferred to the light-sealed imaging cabinet of an IVIS Lumina K machine and positioned in a nose cone in order to maintain anesthesia. Bioluminescent images were acquired using a charge-coupled device camera cooled to −80 °C to achieve maximal sensitivity. Images were acquired at 10 min post substrate injection with an exposure time of 20 s, medium binning, F/Stop = 1, and EM gain off.

### 4.11. Definition of Control

In Figure 4 and Figure 6, there are “0 s” treatments, which represent no µCAP treatment used as control.

### 4.12. Statistical Analysis

Results were plotted using Origin 8 as mean ± standard deviation. Student *t*-test was used to check the statistical significance (* *p* < 0.05, ** *p* < 0.01, *** *p* < 0.001).

## 5. Conclusions

In summary, the effect of a newly developed µCAP on glioblastoma both in vitro and in vivo has been demonstrated. A variety of diagnostics tools were applied to the µCAP, including optical emission spectroscopy, microwave scattering, and potential measurement, which supplied evidence for reactions of plasma-generated species in media and the mouse brain. The µCAP direct treatment has a stronger effect than the indirect treatment due to the synergetic effect of short- and long-lived species, while a strong effect of indirect µCAP treatment on cancer cells is likely attributed to the action of long-lived species. The µCAP is safe for mice and suppresses tumor growth in the mouse brain. These initial observations establish µCAP as a potentially useful ablative therapy in glioblastoma.
